# Combined brain-derived neurotrophic factor and neurotrophin-3 treatment is preferred over either one separately in the preservation of the auditory nerve in deafened guinea pigs

**DOI:** 10.3389/fnmol.2022.935111

**Published:** 2022-09-26

**Authors:** Henk A. Vink, Dyan Ramekers, Hans G. X. M. Thomeer, Huib Versnel

**Affiliations:** ^1^Department of Otorhinolaryngology and Head & Neck Surgery, University Medical Center Utrecht, Utrecht University, Utrecht, Netherlands; ^2^UMC Utrecht Brain Center, University Medical Center Utrecht, Utrecht University, Utrecht, Netherlands

**Keywords:** neurotrophin, hearing loss, neuroprotection, neurodegeneration, cochlear implant, cochlea, eCAP

## Abstract

Severe hearing loss or deafness is often caused by cochlear hair cell loss and can be mitigated by a cochlear implant (CI). CIs target the auditory nerve, consisting of spiral ganglion cells (SGCs), which degenerate gradually, following hair cell loss. In animal models, it has been established that treatment with the neurotrophins brain-derived neurotrophic factor (BDNF) and neurotrophin-3 (NT-3) reduce SGC degeneration. In this study, we aimed to investigate whether treatment with both BDNF and NT-3 (Cocktail) is superior to treatment with each neurotrophin separately regarding cell preservation and neural responsiveness to electrical stimulation. To this end, deafened guinea pigs received neurotrophic treatment in their right ear *via* a gelatin sponge on the perforated round window membrane, followed by cochlear implantation 4 weeks later in the same ear for electrophysiological recordings to various stimulation paradigms. Normal-hearing and deafened untreated guinea pigs were included as positive and negative controls, respectively. Substantial SGC loss occurred in all deafened animals. Each of the neurotrophic treatments led to enhanced SGC survival mainly in the basal turn of the cochlea, gradually decreasing toward the apex. The Cocktail treatment resulted in the highest SGC survival in the treated ear, followed by BDNF, with the least protection of SGCs following NT-3 treatment. Survival of the SGC’s peripheral processes (PPs) followed the same trend in response to the treatment. However, survival of SGCs and PPs in the contralateral untreated ears was also highest in the Cocktail group. Consequently, analysis of the ratio between the treated and untreated ears showed that the BDNF group, which showed low SGC survival in the untreated ear, had the highest relative SGC survival of the three neurotrophin-treated groups. Neurotrophic treatment had positive effects in part of the electrically evoked compound action-potential recording paradigms. These effects were only observed for the BDNF or Cocktail treatment. We conclude that treatment with either BDNF or a cocktail of BDNF and NT-3 is preferred to NT-3 alone. Furthermore, since the Cocktail treatment resulted in better electrophysiological responsiveness and overall higher SGC survival than BDNF alone, we are inclined to recommend the Cocktail treatment rather than BDNF alone.

## Introduction

Sensorineural hearing loss is characterized by damage to or loss of cochlear hair cells in the organ of the Corti. A cochlear implant (CI) can partially restore hearing as it takes over the function of the hair cells and directly stimulates the spiral ganglion cells (SGCs), which are the neurons that make up the auditory nerve. However, the SGCs progressively degenerate following hair cell loss, as the organ of Corti is thought to maintain the SGCs using neurotrophic support (Ylikoski et al., 1974; Spoendlin, 1975; Webster and Webster, 1981; Staecker et al., 1996; Ramekers et al., 2012 [Review]; Zilberstein et al., 2012), which may negatively affect hearing with a CI (Seyyedi et al., 2014; Kamakura and Nadol, 2016). It has been shown that treatment with neurotrophic factors prevents the degeneration of SGCs in deafened animals. Brain-derived neurotrophic factor (BDNF) and neurotrophin-3 (NT-3) both occur naturally in the cochlea (Davis, 2003 [Review]; Green et al., 2012 [Review]; Ramekers et al., 2012 [Review]), and exogenous treatment with these neurotrophins yield comparable beneficial effect on improved neural preservation, as shown in numerous studies using animal models (e.g., Ernfors et al., 1996; Miller et al., 1997, 2007; Gillespie et al., 2004; Richardson et al., 2005; Glueckert et al., 2008; Agterberg et al., 2009; Budenz et al., 2015; Havenith et al., 2015; Ramekers et al., 2015a; Sly et al., 2016; Wise et al., 2016; Vink et al., 2020 [all in guinea pig]; Leake et al., 2011, 2019 [cat]; McGuinness and Shepherd, 2005 [rat]). The receptors for both neurotrophins, TrkB for BDNF, and TrkC for NT-3, are reported to be co-expressed in the cochlear SGCs during development (Ylikoski et al., 1993). However, distributions of the Trk receptors along the cochlea have not been quantified. Additionally, BDNF and NT-3 are expressed in the healthy adult cochlea (Johnson Chacko et al., 2017; Schulze et al., 2022) in what is believed to be an opposite gradient from base to apex. Consequently, cells are being more exposed to BDNF in the base and NT-3 in the apex (Davis, 2003 [Review]). Therefore, a combined application of both neurotrophins could arguably lead to superior SGC survival throughout the deafened cochlea from base to apex.

While studies on treatment with the BDNF&NT-3 cocktail demonstrated enhanced neuronal survival *in vitro* (Mou et al., 1997) and *in vivo* (Staecker et al., 1996; Wise et al., 2005; Landry et al., 2011 [all in guinea pig]) and healthy responsiveness *in vitro* (Needham et al., 2012; Wright et al., 2016), the additional value of combining the two neurotrophins relative to the treatment with one of the neurotrophins has rarely been addressed. Mou et al. (1997) reported moderate SGC survival following treatment with NT-3, and higher SGC survival with BDNF treatment in gerbil SGC cultures, while BDNF and NT-3 co-administration resulted in a strong synergistic effect on SGC survival, exceeding the sum of each separate neurotrophin. When applying the neurotrophins using an osmotic pump (first done by Staecker et al., 1996), differences between the single- or cocktail neurotrophin treatment may be obscured due to a ceiling effect, as the treatment would be distributed throughout the cochlea to such an extent that any further degeneration would be prevented. Such an effect is illustrated by studies, in which BDNF treatment *via* an osmotic pump led to substantial SGC survival throughout the cochlea (Agterberg et al., 2009; Ramekers et al., 2015a), which was at the level of the start of treatment. In contrast, BDNF delivery *via* a gelatin sponge on the perforated round window membrane (RWM) led to SGC survival of a lesser magnitude and was limited to the basal turn of the cochlea (Havenith et al., 2015; Vink et al., 2020). While suboptimal for treatment effectiveness, this delivery method is clinically feasible and well-suited to study synergistic effects, as there is ample room for improvement of cellular preservation compared to the treatment with a single neurotrophin.

In addition to cellular survival, electrophysiological measurements can be used to assess neural health following neurotrophic treatment. Applying electrically evoked compound action potentials (eCAPs), several measures have been reported that reflect neural health, including the inter-phase gap (IPG) effect (Prado-Guitierrez et al., 2006; Ramekers et al., 2014, 2015b; Schvartz-Leyzac et al., 2019, 2020; Vink et al., 2020 [all in guinea pig]), recovery measures, and pulse-train responsiveness (Ramekers et al., 2015a,b). Treatment with BDNF has shown improved neural responsiveness in addition to an increase in cellular survival in deafened guinea pigs relative to untreated controls (Ramekers et al., 2015a; Vink et al., 2020). The effect of NT-3 on the various eCAP measures is unknown. Since BDNF and NT-3 have been reported to induce different temporal response patterns *in vitro*, phasic, and tonic, respectively (Adamson et al., 2002), different eCAP outcomes are expected.

In this study, we investigated the protective effect of a cocktail of BDNF and NT-3 in an ototoxically deafened guinea pig model using a gelatin sponge-mediated delivery, in comparison to either neurotrophin separately, in terms of both cell survival and electrophysiological responsiveness. The latter consisted of analyzing the IPG effect, neural recovery (masker-probe paradigm), and responses to high frequent stimulation (pulse-train paradigm). By using both histological and electrophysiological outcome measures, we aimed to yield a comprehensive analysis of the effects of BDNF and NT-3 on the auditory nerve. Specifically, we investigated whether treatment with NT-3 *via* gelatin sponge can lead to more survival beyond the basal turn and whether the treatment with the neurotrophic cocktail leads to superior survival throughout the cochlea, from base to apex, to each neurotrophin alone.

## Materials and methods

### Animals and experimental overview

Fifty-six adult female guinea pigs (Dunkin Hartley; Hsd Poc:DH; 250–350 g) were obtained from Envigo (Horst, Netherlands) and kept under standard laboratory conditions (*ad libitum* access to food and water; 7:00 AM–7:00 PM light-on cycle; temperature 21°C; humidity 60%). Ototoxic deafening was done by systemic co-administration of kanamycin and furosemide. Two weeks later the animals received a small gelatin sponge containing either BDNF (*n* = 12), NT-3 (*n* = 11), or a 1:1 cocktail of both BDNF and NT-3 (Cocktail; *n* = 12) on the RWM of the right cochlea. Six weeks after deafening, an intracochlear electrode array was implanted in the treated ear, with which eCAP recordings were performed. The animals were subsequently sacrificed and both treated and untreated cochleas were harvested and histologically processed. Data from normal-hearing animals (NH; *n* = 9) and sham-treated animals (PBS; *n* = 12), previously published in Vink et al. (2020), were included in the present study as positive and negative control groups, respectively. The aforementioned BDNF-treated animals were reported on previously by Vink et al. (2020), but in the present study, we present more extensive analyses of both histological and electrophysiological data for all groups. All surgical and experimental procedures were approved by the Dutch Central Authority for Scientific Procedures on Animals (CCD: 1150020174315).

### Deafening procedure

Systemic ototoxic deafening has been shown to lead to symmetric bilateral hair cell loss (West et al., 1973; Versnel et al., 2007; Tisi et al., 2022). The animals were anesthetized with an intramuscular injection of dexmedetomidine (Dexdomitor; Vetoquinol, Breda, Netherlands; 0.25 mg/kg) and ketamine (Narketan; Vetoquinol; 40 mg/kg). Pre-operative analgesia (carprofen; Carporal; Dechra/AST Farma, Oudewater, Netherlands; 4 mg/kg) was administered subcutaneously. An eye ointment (Duratears Z; Alcon, Gorinchem, Netherlands) was applied to prevent dehydration damage to the animal’s eyes. Normal-hearing thresholds, <40 dB peak equivalent sound pressure level (SPL), were verified by recording click-evoked ABRs. Normal-hearing animals were subsequently deafened by subcutaneous injection of kanamycin (Sigma-Aldrich, St. Louis, MO, United States; 400 mg/kg), followed by an infusion of furosemide (Centrafarm, Etten-Leur, Netherlands; 100 mg/kg) into the surgically exposed external jugular vein. Lidocaine (Xylocaine 1% with adrenaline; AstraZeneca B.V., Zoetermeer, Netherlands) was used as a local anesthetic. The wound was subsequently sutured shut, and atipamezole (Atipam, Dechra Pharmaceuticals PLC, Northwich, United Kingdom; 1 mg/kg) was administered intramuscularly to antagonize the dexmedetomidine-induced anesthesia, after which the animals were left to recover in a pre-heated cage.

### Gelatin sponge-mediated neurotrophin delivery

Two weeks after the deafening procedure, the animals were anesthetized again with an intramuscular injection of dexmedetomidine (0.25 mg/kg) and ketamine (40 mg/kg). Carprofen (4 mg/kg) was subcutaneously injected, as pre-operative analgesia, together with the non-ototoxic antibiotic enrofloxacin (Baytril, Bayer AG, Leverkusen, Germany; 5 mg/kg). Following these injections, eye ointment was applied to prevent eye damage during the procedure. Click-evoked ABRs were recorded to verify that the animals were sufficiently deafened: a threshold shift of >50 dB was used as an inclusion criterion. A C-shape incision was made behind the right ear of the animal, to expose the auditory bulla. A small hole was hand-drilled in the bulla to allow access to the RWM of the cochlea, which was subsequently perforated (Havenith et al., 2015; Vink et al., 2020). An absorbable gelatin sponge (Spongostan™ Dental; Ethicon, Somerville, NJ, United States; fully absorbed in 4 to 6 weeks) was pressed flat (∼1 mm thick) to easily punch a small (∼1 mm^3^) cylinder with a custom-made 1-mm-diameter cylindrical punch. Subsequently, 3 μl of the treatment solution was pipetted adjacent to the sponge. The sponge cylinder was then pushed into the treatment solution until the sponge was saturated and the solution almost entirely soaked up. The saturated sponge was then placed into the RWM niche with a micro pick instrument, pressing against the perforated RWM. As the sponge sticks to wet surfaces, contact with the perforated RWM also ensured that the sponge would remain in place for the duration of the experiment. Note: the gelatin sponge absorption time of 4 to 6 weeks corresponded with the duration of the experiment following the sponge application. Consequently, the sponge was still visible on the RWM in only a few animals at the time of cochlear implantation. Importantly, in no instance was the gelatin sponge observed in another location within the bulla. The contralateral (left) cochlea remained untouched and was used as a within-subject negative control. Each animal was quasi-randomly assigned to one of the treatment groups prior to this surgical procedure. The treatment solution consisted of PBS with 15% dimethyl sulfoxide (DMSO) with either BDNF (PeproTech, London, United Kingdom; 6.67 μg/μl [247 μM]), NT-3 (PeproTech, London, United Kingdom; 6.67 μg/μl [245 μM]) or a cocktail of the two (3.33 μg/μl [123 μM] of BDNF and 3.33 μg/μl [122 μM] of NT-3). This means that the gelatin sponge was loaded with either 20 μg BDNF, 20 μg NT-3, or 20 μg of the combined neurotrophins. It is not known whether the neurotrophins are released within a few days or over the course of several weeks. In the former case, it is relevant to consider that the neuroprotective effect is assumed to persist after cessation of delivery (Ramekers et al., 2015a). DMSO was added to the solution to allow for a fair comparison of the results between the BDNF, and the NT-3 and Cocktail groups, as DMSO was a necessary addition in a study, from which the BDNF data was derived (see Vink et al. [2020] for details). In that study, we demonstrated in control animals that the addition of 15% DMSO did not affect cochlear histology. The hole in the bulla was sealed with dental cement (GC Fuji PLUS; GC Corporation, Tokyo, Japan) and the wound was sutured shut. The animals were given intramuscular atipamezole (1 mg/kg) and placed in a pre-heated cage to recover.

### Cochlear implantation

Four weeks after the gelatin sponge placement, the animals were anesthetized with an intramuscular injection of Hypnorm^®^ (Vetapharma Ltd, Leeds, United Kingdom; 0.5 ml/kg), followed by exposure to 2% isoflurane in a gas mixture of (1:2) O_2_ and N_2_O *via* a mouth cap. Subcutaneous injection of glycopyrronium (Robinul; Chiesi Pharmaceuticals GmbH, Vienna, Austria; 0.02 mg/kg) was administered to prevent aspiration during the experiment. An incision was made on the head to expose the skull. Two transcranial screws were placed 1 cm bilateral to bregma, which were used as reference electrodes for eCAP stimulation and recording. A tracheostomy was then performed before the animal was transported to a heating pad (37°C), on which the animals were artificially ventilated (Amsterdam infant ventilator mk3, Hoekloos, Schiedam, Netherlands; 45–50 cycles/min respiration rate, 1.8–2.3 kPa) through the tracheostomy with a gas mixture of (1:2) O_2_ and N_2_O and 1–1.5% isoflurane for the remainder of the experiment. Because of the long duration of the eCAP recording session of up to 6 h, continuous gas anesthesia was preferred over repeated injection anesthesia. The right bulla was reopened by removing the dental cement and a 0.5-mm cochleostomy was hand-drilled into the cochlea within 1 mm from the RWM. A custom-made four-contact electrode array (MED-EL GmbH, Innsbruck, Austria) was inserted ∼4 mm into the scala tympani, including all four contacts (situated in the first 3 mm, with a 1-mm distance between each contact). The most basal electrode was ∼2 mm and the most apical electrode was ∼5 mm from the round window.

### Auditory brainstem response recordings

Click-evoked ABRs were recorded before the deafening procedure and prior to the gelatin sponge placement. Three subcutaneous needle electrodes were used for these recordings: the active electrode was placed behind the right ear, the reference electrode was placed on the skull, and the ground electrode was placed in the hind limb. A Blaupunkt speaker [PCxb352; 4 Ω; 30 W, Blaupunkt (International) GmbH & Co. KG, Hildesheim, Germany], set at a 10-cm distance from the right ear presented short broadband acoustic clicks (20 μs monophasic rectangular pulses; inter-stimulus interval 99 ms) synthesized and attenuated by a TDT3 system (Multi-I/O processor RZ6; Tucker-Davis Technologies, Alachua, FL, United States), and a Princeton Applied Research (Oak Ridge, TN, United States) 5113 pre-amplifier (×5,000; bandpass filter 0.1–10 kHz) was used. The sound levels were calibrated by a sound level meter (Brüel and Kjaer, 2203) and a 1″ condenser microphone (Brüel and Kjaer, 4132). The recordings were digitized by the TDT3 system (100 kHz sampling rate, 24-bit sigma-delta converter) and stored for offline analysis. Hearing thresholds were determined by presenting a sound level of 110 dB peak equivalent SPL, which was decreased in steps of 10 dB until no response was observed. The threshold was defined as the interpolated sound level, at which the ABR N_1_-P_2_ peak was 0.3 μV.

### Electrically-evoked compound action potential recordings and analysis

A MED-EL PULSAR CI stimulator (MED-EL GmbH, Innsbruck, Austria) was used for eCAP recordings. The CI was controlled by a PC *via* a Research Interface Box 2 (RIB2; Department of Ion Physics and Applied Physics, University of Innsbruck, Innsbruck, Austria) and a National Instruments data acquisition card (PCI-6533, National Instruments, Austin, TX, United States). Stimulation/recording paradigms were executed using MATLAB (version 7.11.0; Mathworks, Natick, MA, United States). In most cases, the most apical electrode of the four-electrode array (situated at approximately 4 mm inside the cochlea) was used for monopolar stimulation and the most basal one was used for recording. Three different eCAP recording protocols were utilized in this study, previously described by Ramekers et al. (2014: single-pulse protocol; 2015b: masker-probe protocol and pulse-train protocol). Biphasic current pulses were presented with alternating polarity to reduce the stimulation artifact, and the responses to 50 pairs (100 pairs for the masker-probe protocol) of these stimuli were averaged. The experimenters were blinded during the offline analyses of the eCAP recordings.

#### Single-pulse recordings

**FIGURE 1:**
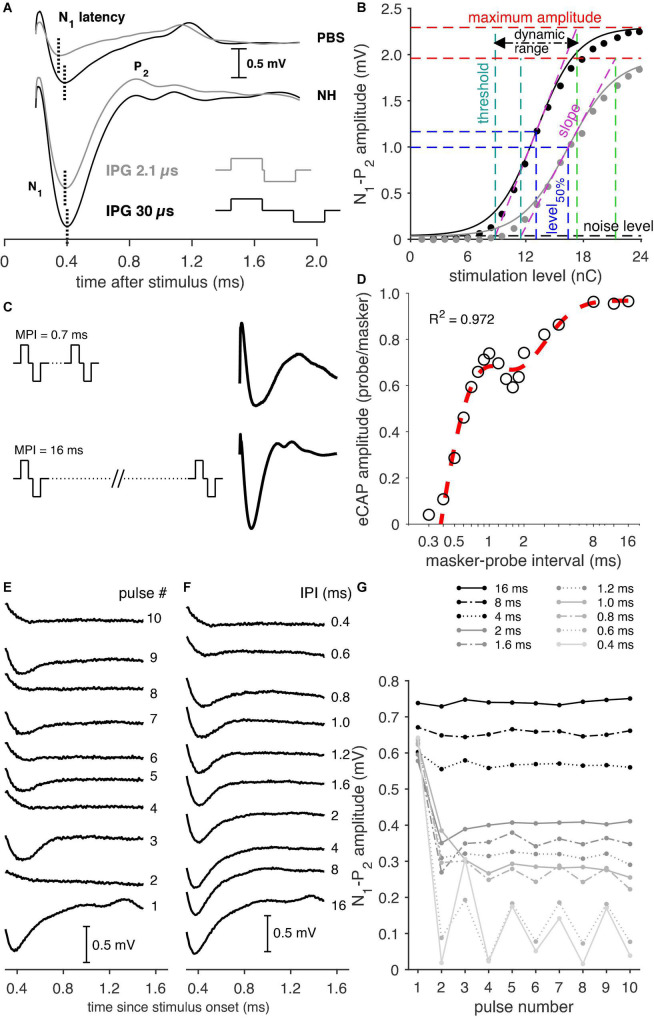
Examples of the various eCAP recording paradigms. **(A)** Examples of eCAPs from a PBS animal (top) and a normal-hearing (NH) animal (bottom) for both an IPG of 2.1 and 30 μs (pulse shapes illustrated in the lower right corner), with the indication of the N_1_ latency. **(B)** Input–output curves of an NH animal for an IPG of 2.1 (gray markers) and 30 μs (black markers), with corresponding sigmoid fitting (solid lines) and the derived eCAP measures. **(C)** Examples of eCAP recordings following a short (0.7 ms) masker-probe interval (MPI) and the longest (16 ms) MPI from a normal-hearing animal. **(D)** Example of an eCAP amplitude recovery function from an NH animal including the double exponential fit (red trace). **(E)** eCAP recordings from a Cocktail animal to the first ten pulses of a pulse train with an inter-pulse interval (IPI) of 0.6 ms. Note the alternating pattern in the eCAP waveform, with higher amplitudes following odd-numbered pulses and lower amplitudes following even-numbered pulses. **(F)** Examples of eCAPs evoked by the tenth pulse at each IPI used in the pulse-train paradigm. Note the gradual decrease of eCAP amplitude and increase of N_1_ latency with shorter IPIs. **(G)** The eCAP N_1_-P_2_ amplitudes in response to each of ten pulses for every IPI (0.4–16 ms).

For the single-pulse recordings, the phase duration (PD) was 30 μs and the IPG of the stimuli was either 2.1 or 30 μs. As shown in Figures 1A,B, increasing the IPG leads to changes in the eCAP, e.g., an increase in eCAP amplitude and latency, and a decrease in the threshold. The pulse for both IPGs was presented at 20 stimulation levels, typically ranging from 1.2 to 24 nC, with a maximum stimulation level ranging from 21 to 28.5 nC (21: *n* = 3 animals; 24: *n* = 31; 25.5: *n* = 12; 27: *n* = 8; 28.5: *n* = 1). For the eCAP analysis, an input-output function was created by plotting the eCAP amplitude, defined by the difference in voltage between P_2_ and N_1_ peaks, against the stimulation level. This function was fitted with a Boltzmann sigmoid with the following Equation (1) as in Ramekers et al. (2014).


(1) $$V_{e\,C\,A\,P}=A+\frac{B}{1+e^{-\frac{I-C}{D}}},$$

*V*_eCAP_ is the eCAP amplitude in mV, *I* represent the stimulation current in μA, and *A–D* is the fitting parameters. From these parameters, several measures can be derived, as shown in Figure 1B. These are the maximum eCAP amplitude (B), the stimulation current level, at which the amplitude is 50% of its maximum (C; level_50%_), the slope at C (B/4D), the stimulation threshold (C-2D), and the range in stimulation level between threshold and maximum amplitude (4D; dynamic range). These measures were supplemented with the N_1_ peak latency, averaged over the three highest current levels.

#### Masker-probe recordings

For the masker-probe protocol, two pulses were presented: the first acted as the masker and the second as the probe to measure the recovery and facilitation characteristics of the auditory nerve (Morsnowski et al., 2006; Ramekers et al., 2015b). The masker-probe interval (MPI) was varied between 0.3 and 16 ms in 18 steps. This range was chosen to (1) allow for a long enough interval for the longest MPI (16 ms) to avoid the effect of refractoriness on the probe and (2) for the shortest MPI (0.3 ms) to be shorter than the absolute refractory period to extinguish the neural response to the probe stimulation. The PD of both pulses was 50 μs and the IPG was 30 μs. The pulses were presented in 10 stimulation level steps, the range of which was typically the same as used for the single-pulse paradigm. A ∼6 ms eCAP recording was constructed from five ∼1.7-ms recordings by stepwise increments of the measurement delay after stimulus onset; see Figure 1C for examples of eCAP recordings following long and short MPIs. The masker-evoked and the probe-evoked eCAPs were recorded separately and consecutively, because the implant was not able to perform continuous recording. For MPIs shorter than the recording window (<6 ms), the masker-evoked activity was subtracted from the probe-evoked responses, as the response to the masker was present in the probe-evoked recordings.

For each animal, the probe-evoked eCAP amplitudes, recorded only at the highest stimulation level (between 21 and 28.5 nC, as mentioned in Section “Single-pulse recordings”), were normalized to the amplitude of the corresponding masker-evoked eCAP. Subsequently, the recovery function, i.e., the normalized eCAP amplitude per MPI, for every animal was fitted with a double exponential function as in Ramekers et al. (2015a,b), as exemplified in Figure 1D:


(2) $$e\,C\,A\,P=A\cdot \left(1+c\cdot e^{-\frac{M\,P\,I-t_{0}}{\tau_{A}}}\right)\cdot \left(1-e^{-\frac{M\,P\,I-t_{0}}{\tau_{B}}}\right),$$

in which *eCAP* is the normalized probe-evoked eCAP amplitude for a masker-probe interval *MPI*, *A* is the normalized maximum amplitude of the probe-evoked eCAP, *c* is a constant defining the ratio between the two exponential components, *τ_*A*_* represents the recovery time constant of the first exponential, *τ_*B*_* represents that of the of second exponential, and *t*_0_ is the absolute refractory period. A double exponential fit was chosen to account for the non-monotonic course of the recovery functions (Figure 1D). The second exponential with time constant *τ_*B*_* describes the actual recovery from refraction, while the first exponential with time constant *τ_*A*_* describes a facilitatory process giving rise to the local maximum in the recovery function at approximately 1 ms MPI (see also Ramekers et al., 2015b). The N_1_ latency, averaged over the three highest stimulation levels (typically 19.2, 21.6 and 24 nC), was used as an additional measure.

#### Pulse-train recordings

In the pulse-train protocol series of identical pulses (PD and IPG both 30 μs) were presented at the highest current level used for the single-pulse recordings. The responses to each pulse in a ten-pulse pulse train and to the last ten pulses of a 100-ms long pulse train were recorded stepwise. This allowed us to capture both the rapid initial effects of neural fatigue and the more adapted steady-state responses at the end of the stimulation. The inter-pulse intervals (IPIs) ranged from 0.4 to 16 ms – examples, of which are shown in Figures 1F,G – and were chosen as such to avoid refractoriness or adaptation at the longest II, but to allow for a gradual reduction of the eCAP amplitude due to, e.g., refractoriness, neural fatigue, and/or adaptation for shorter IPIs.

The pulse-train eCAP recordings were analyzed as described by Ramekers et al. (2015a,b). In short, an alternating pattern can be observed for the shorter IPIs (see Figure 1G). To quantify this pattern, we applied Fourier analysis, which yielded a measure of amplitude modulation depth as a percentage of the maximum eCAP amplitude for each individual animal. To avoid interference of this alternating pattern by the initial drop in eCAP amplitude after the first pulse, the modulation depth of this alternating pattern was determined for the last six pulses only (see 0.4–0.8-ms IPI in Figure 1G). The N_1_ peak latency of both the first and last ten pulses averaged over each ten, was used as an additional measure. Note that, of the first ten pulses, for both the Fourier analysis and the mean latency, only the final six of the ten pulses were used.

### Histological processing

Both the treated and untreated ears of each animal were harvested, following its termination immediately after the eCAP recordings. The cochleas were then fixated as described by De Groot et al. (1987). In short, the cochleas were fixated by an intra-labyrinthine infusion with a fixative of 3% glutaraldehyde, 2% formaldehyde, 1% acrolein, and 2.5% dimethyl sulfoxide (DMSO) in a 0.08-M sodium cacodylate buffer. Following fixation, the cochleas were decalcified, post-fixated, and embedded in Spurr’s low-viscosity resin. Staining was done using 1% methylene blue, 1% azur B, and 1% borax in distilled water. The cochleas were then divided into two halves along a standardized midmodiolar plane and re-embedded in fresh resin. From one of the halves, two semithin (1 μm) sections were cut at a 60-μm interval, so that the possibility of a single SGC being present in two subsequent sections was avoided. From the same half, semithin transverse sections were made from the osseous spiral lamina (OSL) for the basal, middle, and apical turns for analysis of peripheral processes (PPs; as described by Ramekers et al., 2020).

### Spiral ganglion cell and peripheral process analysis

**FIGURE 2:**
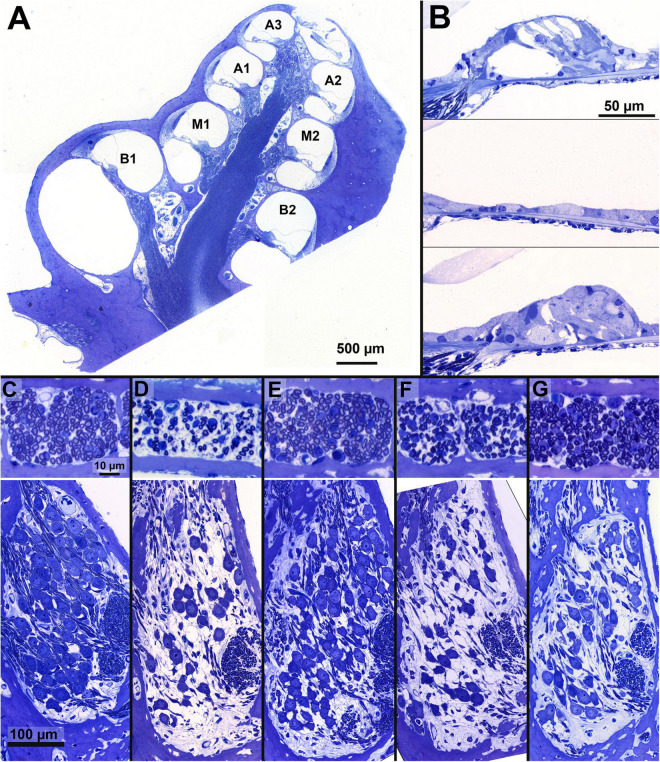
Examples of cochlear micrographs. **(A)** A cross-section of a guinea pig cochlea along a midmodiolar plane, with the seven cochlear locations (semi-turns), B1-A3 from base to apex. **(B)** Examples of the organ of Corti containing the cochlear hair cells, with an NH example (top, M1), and two examples following ototoxic deafening: a completely degenerated (middle, M1) and a collapsed and degenerating organ of Corti (bottom, M2). **(C–G)** Examples of a cross-section of peripheral processes in the osseous spiral lamina of the basal turn (in between B1 and B2; top) and Rosenthal’s canal from the upper basal turn (B2) containing the SGC somata (bottom) from a single animal per group, with a normal-hearing example in panel **(C)**. The average PP and SGC survival relative to NH, of the examples are given in percentages: **(D)** PBS: SGC 49%, PP 37%; **(E)** BDNF-treated: SGC 65%, PP 81%; **(F)** NT-3-treated: SGC 37%, PP 75% and **(G)** a Cocktail-treated animal: SGC 78%, PP 85%.

Micrographs of SGCs and PPs were taken with a Leica DFC450 digital camera mounted on a Leica DMRA light microscope (Leica Microsystems GmbH, Wetzlar, Germany). For SGC analysis micrographs of Rosenthal’s canal of the cochlear regions B1, B2, M1, M2, A1, A2, and A3 (Figure 2A) were obtained from the two semithin sections using a 40× oil immersion objective (Leica Microsystems GmbH). In 18 out of 47 deafened animals, it was not possible to acquire the A3 region of both the treated and contralateral ear. For all other cochlear regions, bilateral micrographs could be obtained for at least 43 of 47 animals. Therefore, analyses of the A3 region were omitted from the present study. For the remaining regions, SGC packing density (the number of cells divided by cross-sectional area, in SGC/1000 μm^2^) was established using ImageJ (version 1.52a; National Institutes of Health, Bethesda, MD, United States), as described previously (Van Loon et al., 2013; Kroon et al., 2017). In short, this was done by outlining the bony boundaries of Rosenthal’s canal and subsequently quantifying all SGCs within it, regardless of nucleus presence or cell size. Only the SGCs with a visible nucleus were outlined to establish their size to determine the mean perikaryal area for each cochlear location. The resulting packing density was corrected for the mean perikaryal area (Coggeshall and Lekan, 1996; Van Loon et al., 2013), as the SGC size affects the likelihood of detecting the cell. This correction was made using the following equation, as described in Van Loon et al. (2013):


(3) $$b_{\text{cor}}=b\cdot \sqrt{\frac{a_{\text{n}}}{a}}$$

In which *b*_*cor*_ is the corrected packing density, *b* is the originally determined packing density, *a*_*n*_ is the mean perikaryal area of the SGCs in the normal-hearing group, and *a* is the mean perikaryal area of the SGCs in the corresponding individual cochlear location. Finally, *b*_*cor*_ was averaged across both semithin sections. Following the SGC analyses, the organ of Corti of each cochlear location was inspected in one of the mid-modiolar sections, from which both inner hair cells (IHCs) as outer hair cells (OHCs) were quantified. These quantifications were used as an estimation of the remaining hair cells following the deafening procedure.

For the PPs, micrographs were obtained from the basal (B), middle (M), and apical (A) OSL sections using a 63× oil immersion objective (Leica Microsystems GmbH). PP packing densities (as the number of PPs divided by the OSL cross-sectional area, in PPs/1000 μm^2^) for each region were obtained by outlining the bony boundaries of the OSL and quantifying the number of PPs inside.

The packing density outcomes for both the SGC and PP analyses were normalized to mean packing densities from cochleas of NH animals to allow for a 1:1 comparison of SGC and PP preservation. The ears used for this normalization were the non-implanted ears from NH animals that received unilateral cochlear implantation (obtained from Ramekers et al., 2015a), to circumvent possible effects of cochlear implantation or electrical stimulation. These normalization values for SGCs were: 1.420 (B1); 1.744 (B2); 1.714 (M1); 1.632 (M2); 1.675 (A1); and 1.543 (A2) cells/1,000 μm^2^. Normalization was performed by dividing the packing density of each cochlear location for each individual animal by the mean NH packing density of the corresponding cochlear location. These normalization values for the PPs were 82 (B), 69 (M), and 60 (A) PPs/1,000 μm^2^. Histological analyses were performed by several experimenters, who were randomly assigned to the histological sections, regardless of the experimental group, animal, or treated or untreated ear. All experimenters were blinded during all the histological analyses.

### Statistics

Linear mixed model (LMM) analyses were applied on the histological data under the assumption of compound symmetry for the following: (1) the treated right cochlea, (2) untreated left cochlea, or (3) the log_2_-transformed ratio of the treated/untreated ears with cochlear location as a covariate and treatment group as a factor. The cochlear location in these analyses was the distance relative to the B1 region, as previously described by Van Loon et al. (2013). Following the LMM, an analysis of variance (ANOVA) was performed on a subset of the data, specified in the appropriate section, with subsequent *post hoc* testing by means of Tukey’s honest significant difference test (HSD). For the eCAP data, group differences were evaluated using an ANOVA, followed by Tukey’s HSD for *post hoc* testing. A Pearson’s correlation coefficient was used to determine the relationship between the various eCAP characteristics and the corresponding histological data. In addition, a stepwise multiple regression analysis was performed on specific eCAP data to determine whether they were dependent on SGC packing density and size could be used as a predictor. All statistics were performed in SPSS version 26 for Windows (IBM Corp, Armonk, NY, United States).

## Results

### Deafening results and animal inclusion

All animals were considered to be sufficiently deafened following the ototoxic procedure, with an average ABR threshold shift of 76 dB (ranging from 58 to 87 dB). This was further corroborated by loss of hair cells in the collapsed organ of Corti following ototoxic deafening, as exemplified in Figure 2B (middle and bottom). In addition, hair cell quantification (Table 1) was in line with symmetric bilateral deafening as remaining hair cell survival was similar between the right treated and left untreated cochleas for all experimental groups. Nor did the remaining hair cells lead to any residual hearing, as indicated by the ABR threshold shifts. Note that the mention of ‘experimental groups’ refers to the four deafened groups: PBS, BDNF, NT-3, and Cocktail, whereas ‘neurotrophin-treated groups’ refers only to the latter three.

**TABLE 1:** Mean percentage of remaining inner hair cells (IHCs) and outer hair cells (OHCs) of both the treated and untreated cochleas, and the mean ABR threshold shift, following ototoxic deafening.

Group	IHC (% re NH)		OHC (% re NH)		Mean ABR
	Treated	Untreated	Treated	Untreated	Threshold shift (dB)
BDNF	23.8 (7.3)	29.0 (5.8)	7.7 (3.2)	2.8 (1.5)	80 (0.5)
NT-3	12.8 (6.1)	5.6 (2.4)	1.4 (0.7)	0.0 (0.0)	76 (1.3)
Cocktail	22.0 (4.9)	17.3 (4.6)	2.0 (1.3)	1.7 (0.9)	76 (2.3)
PBS	27.8 (6.9)	32.4 (7.3)	27.0 (5.7)	27.4 (5.7)	72 (2.4)

SEM is included in brackets for each mean.

From the PBS group, two animals died from respiratory complications prior to the masker-probe and pulse-train eCAP paradigms. In addition, due to a human error, it was not possible to record the pulse-train eCAPs in one animal from the PBS group. Finally, one BDNF-treated animal suffered fatal respiratory complications before cochlear implantation (as mentioned in Vink et al., 2020). As we decided to only include animals in the present study, for which both histological and electrophysiological data was available, this animal was excluded from the present study. These exclusions lead to the following group sizes: BDNF, *n* = 11; NT-3, *n* = 11; Cocktail, *n* = 12; PBS, *n* = 12 for histological analysis and single pulse eCAP recordings, *n* = 10 for the masker-probe, and *n* = 9 for the pulse-train eCAP recordings.

### Histological results

Examples of cochlear micrographs from the basal turn are shown in Figures 2C–G, with cross-sections of the OSL containing PPs (top, location B1-B2) and Rosenthal’s canal containing SGCs (bottom, location B2) for each of the five groups. In these examples, the number of PPs and SGCs decreased drastically after 6 weeks of deafness (Figure 2D), as compared to the NH group (Figure 2C). After 6 weeks of deafness with neurotrophic treatment, survival of both SGCs and their PPs was generally substantially higher (Figures 2E–G; BDNF, NT-3, and Cocktail, respectively) than the PBS control group.

#### Spiral ganglion cells

**FIGURE 3:**
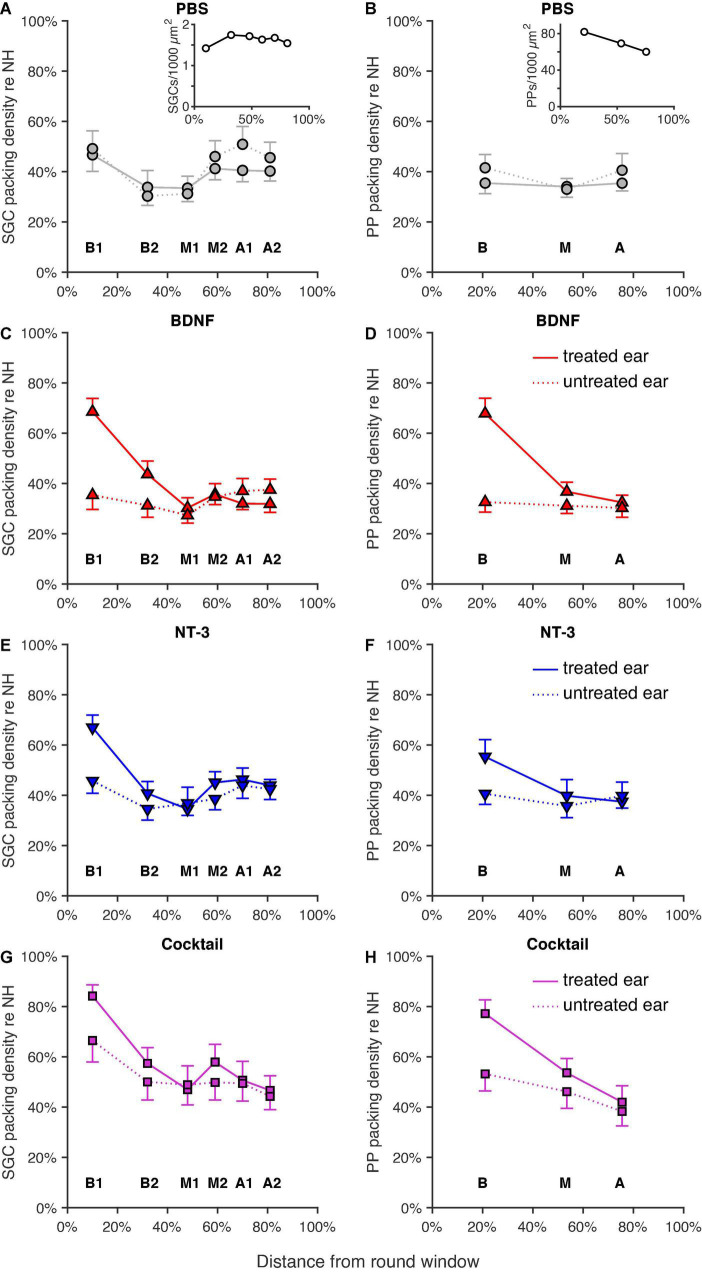
Normalized SGC and PP packing densities as a function of cochlear location as a relative distance to the round window, with 0% as the round window membrane and 100% being the helicotrema. Group means of SGC (left column) and PP (right column) packing densities for both the treated right ears (solid lines) and the untreated left ears (dotted lines) are shown for **(A,B)** the PBS group (*n* = 12; *n* = 11 for location A in **B**), the insets represent the absolute SGC **(A)**, and PP **(B)** packing densities for the NH animals, **(C,D)** the BDNF-treated group (*n* = 11), **(E,F)** the NT-3-treated group (*n* = 11; *n* = 10 for location B1 in **F**), and **(G,H)** the Cocktail-treated group (*n* = 12; *n* = 10 for location B1 in **G**; *n* = 11 for location A in **H**). Error bars represent SEM.

The mean normalized SGC packing densities (see Section “Spiral ganglion cell and peripheral process analysis”) in each cochlear location (expressed in % relative distance from the RWM; see Van Loon et al., 2013, their Figure 2) for the four experimental groups are shown in Figures 3A,C,E,G. In all neurotrophin-treated groups, survival appeared to have increased mainly in the basal turns of the cochlea, but with increased survival in the M2 region, as well for the NT-3 and Cocktail treatment groups.

Indeed, a linear mixed model analysis revealed a statistically significant treatment effect [*F*_(3,78.5)_ = 5.6, *p* = 0.0015] with both the BDNF and Cocktail treatment resulting in significantly more SGC survival than in the PBS control group [*t*_BDNF(77.0)_ = 2.0, *p* = 0.051; *t*_Cocktail(80.1)_ = 4.1, *p* < 0.001] and the NT-3 treatment group approaching a significant effect [*F*_NT–3(77.0)_ = 1.8, *p* = 0.075]. Additionally, a significant interaction effect between treatment and cochlear location [*F*_(3,84.4)_ = 4.8, *p* = 0.0040] was observed, which was statistically significant for each neurotrophin-treated group (*p*_BDNF_ = 0.0010; *p*_NT–3_ = 0.029; *p*_Cocktail_ = 0.0029). As the treatment effect was mainly observed in the basal turn of the cochlea, and a subtle effect is visible from the M1 region onward, we averaged the results from B1 and B2 into “B”, and M2, A1 and A2 into “M2A” for *post hoc* testing. An ANOVA on B and M2A revealed a statistically significant difference in SGC packing density between groups [*F*_B(3,40)_ = 5.0, *p* = 0.0050; *F*_M2A(3,42)_ = 3.2, *p* = 0.033]. A follow-up Tukey’s HSD analysis (Table 2) revealed that following treatment with the Cocktail, SGC survival was significantly higher than in the PBS group in the basal turn (*p* = 0.0022). For M2A, more SGC survival was observed in the Cocktail treatment group than in the BDNF treatment group (*p* = 0.022).

**TABLE 2:** *P*-values from Tukey’s pairwise comparison following the ANOVA as *post hoc* of the linear mixed model, performed on the normalized packing densities of both SGCs and PPs for the treated ear.

Comparison		SGC: B	SGC: M2A	PP: B	PP: M
BDNF–NT-3	*p*	0.99	0.25	0.43	0.97
BDNF–Cocktail*	*p*	0.38	**0.022**	0.65	0.090
BDNF*–PBS	*p*	0.15	0.63	**0.0012**	0.98
NT-3–Cocktail*	*p*	0.24	0.70	**0.044**	0.21
NT-3–PBS	*p*	0.27	0.88	0.077	0.84
Cocktail*–PBS	*p*	**0.0022**	0.27	**<0.001**	**0.032**

The (*) indicates the group with the highest normalized packing density in the comparison yielding a p < 0.05. p-values < 0.05 are shown in bold.

In the contralateral untreated ears (Figures 3A,C,E,G, dotted lines), SGC degeneration appeared to be quite similar between groups except for the Cocktail group, which remarkably showed the highest SGC survival in the untreated ear. This was confirmed with a linear mixed model analysis, indicating an effect of treatment [*F*_(3,64.3)_ = 3.9, *p* = 0.013] in these contralateral ears, with the Cocktail group resulting in a higher packing density than the PBS group [*t*_(64.3)_ = 2.2, *p* = 0.028]. No interaction effect, but approaching significance, between treatment and location was observed [*F*_(3,98.1)_ = 2.3, *p* = 0.078].

#### Peripheral processes

The analyses of PPs (Figures 3B,D,F,H) show a similar picture as that of the SGCs: PP survival following neurotrophic treatment was most prominently present in the basal turn of the cochlea. The Cocktail treatment resulted in the largest number of PPs, followed by BDNF, and then NT-3. An LMM analysis on these results indicated a treatment effect [*F*_(3,57.2)_ = 11.6, *p* < 0.001], and an interaction effect between treatment and location [*F*_(3,80.5)_ = 5.6, *p* = 0.0014]. A follow-up ANOVA revealed that this treatment effect was significantly present in both the basal and middle turn of the cochlea [*F*_B(3,42)_ = 10.5, *p* < 0.001; *F*_M(3,42)_ = 3.2, *p* = 0.032]. *Post hoc* testing (Table 2) revealed that in the basal turn, treatment with both BDNF and the Cocktail led to significantly more PP survival than in the PBS group (*p*_BDNF–PBS_ = 0.0012; *p*_Cocktail–PBS_ < 0.001), and treatment with the Cocktail led to significantly more PP survival than with NT-3 alone (Tukey’s HSD, *p* = 0.044). In the middle turn, only the Cocktail treatment led to significantly more PP survival than in the PBS group (Tukey’s HSD, *p* = 0.032).

In the untreated ears (Figures 3B,D,F,H, dotted lines), similar to the SGC data, there was more PP survival in the Cocktail treatment group than in the other experimental groups. Additionally, the data from this group appeared to follow the same pattern as observed in the treated ear: the most PP survival in the basal turn, but reduced survival toward the apex. An LMM analysis on these ears revealed a treatment effect [*F*_(3,54.9)_ = 3.3, *p* = 0.026], with the Cocktail treatment leading to significantly higher PP survival than the PBS control treatment [*t*_(54.8)_ = 2.2, *p* = 0.036]. No interaction between treatment and location was observed [*F*_(3,85.5)_ = 0.67, *p* = 0.57].

#### Spiral ganglion cell and peripheral process data, treated/untreated ratio

**FIGURE 4:**
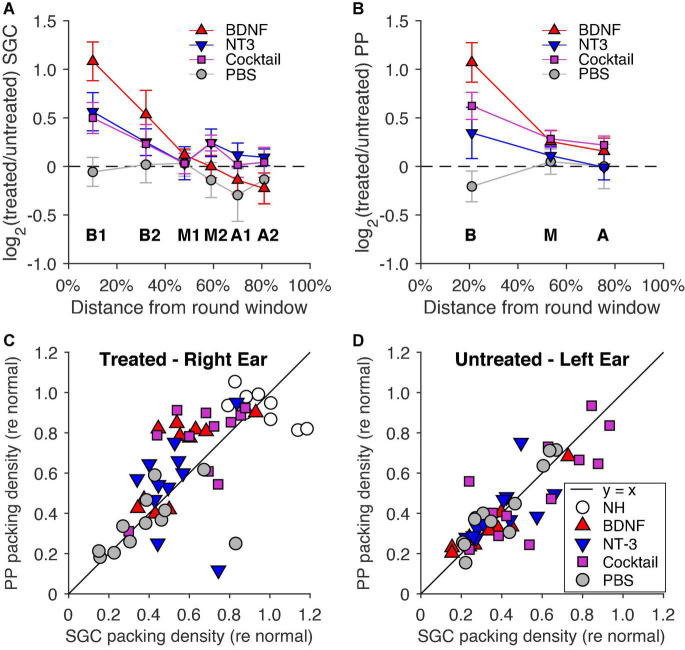
Log_2_ transformed ratio between the right (treated) and left (untreated) ears for **(A)** SGC packing density and **(B)** PP packing density as a function of cochlear location relative to the round window, with 0% as the round window and 100% as the helicotrema. The dashed line indicates equal packing densities between the treated and untreated ears. PBS, *n* = 12 (*n* = 11 for location A in **B);** BDNF, *n* = 12; NT-3, *n* = 11 (*n* = 10 for location B1 in **A**); Cocktail, *n* = 12 (*n* = 10 for location B1 in **A**; *n* = 11 for location A in **B**). Error bars represent SEM. Normalized basal packing densities of PPs are shown as a function of normalized SGC packing density for **(C)** the right (treated) ear and **(D)** the left (untreated) ear. The black *y* = *x* line represents the 1:1 ratio of PP: SGC packing density.

For the most comprehensive analysis of treatment efficacy on SGC histology, we compared packing densities in the treated ear to that of the untreated ear, using the latter as an internal negative control for each animal individually. To do this, we calculated the ratio of the packing density for every cochlear location for each animal between the treated right ear and the untreated left ear. This ratio was subsequently log_2_ transformed to allow for a similar scale for positive and negative ratios and to have ‘0’ reflect equal survival. Figure 4 shows this log_2_ transformed ratio for each group, with Figure 4A showing the SGC data and Figure 4B showing the PP data.

Figure 4A shows that the BDNF group had the highest relative SGC survival of all three treatment groups, with NT-3 and Cocktail groups having a highly similar survival ratio between their treated and untreated ears. An LMM analysis revealed a treatment effect [*F*_(3,94.7)_ = 7.3, *p* < 0.001] and interaction effect between treatment and location [*F*_(3,122.5)_ = 5.4, *p* = 0.0015], with a strongly significant treatment effect for the BDNF-treated animals [*t*_(91.0)_ = 4.6, *p* < 0.001] and with a close-to-significant treatment effect for both NT-3 and Cocktail groups [*t*_NT–3(93.7)_ = 1.9, *p* = 0.058; *t*_Cocktail(96.1)_ = 1.8, *p* = 0.076]. Following the same analysis as on the separate ears, a *post hoc* ANOVA was performed on the transformed treated/untreated ratios of B1 and B2 averaged into “B”, and M2, A1, and A2 averaged into “M2A”. This analysis revealed that the treatment effect was limited to basal turn B [*F*_(3,42)_ = 5.2, *p* = 0.0040], and not present in M2A [*F*_(3,42)_ = 1.4, *p* = 0.24]. A Tukey’s HSD analysis (Table 3) was subsequently performed to reveal that the treatment effect was due to BDNF having a significantly higher ratio than the PBS group (*p* = 0.0021).

**TABLE 3:** *P*-values for Tukey‘s pairwise comparison following ANOVA as *post hoc* of the linear mixed model, performed over the log_2_ transformed ratio between the treated and untreated ears.

Histology		SGC: B	PP:B
BDNF–NT-3	*p*	0.39	0.060
BDNF–Cocktail	*p*	0.09	0.37
BDNF*–PBS	*p*	**0.0021**	**<0.001**
NT-3–Cocktail	*p*	0.87	0.74
NT-3–PBS	*p*	0.14	0.20
Cocktail*–PBS	*p*	0.45	**0.018**

The (*) indicates the group with the highest log transformed packing density ratio in the comparison yielding a p < 0.05. p-values < 0.05 are shown in bold.

In Figure 4B, the PP survival ratio of the treated/untreated ears was the highest for the BDNF treatment group, with a treatment effect visible for the other neurotrophin-treated groups as well. A LMM analysis revealed a treatment effect [*F*_(3,69)_ = 6.7, *p* < 0.001] and an interaction effect between treatment and cochlear location [*F*_(3,81.3)_ = 3.3, *p* = 0.025]. A follow-up ANOVA was performed to assess the treatment effect for each cochlear location. This analysis revealed a difference between treatment groups in the basal turn only [*F*_(3,42)_ = 7.7, *p* < 0.001], with both BDNF and the Cocktail treatment resulting in a higher PP survival ratio than the PBS control group (Tukey’s HSD, *p*_BDNF_ < 0.001; *p*_Cockail_ = 0.018).

#### Peripheral process versus spiral ganglion cell survival

As a final step in the histological analysis, Figures 4C,D show the normalized PP survival as a function of normalized SGC survival of all individual animals from each experimental group in the basal cochlear turn for both the treated (Figure 4C) and untreated (Figure 4D) ears. To reiterate, this normalization was performed with data from cochleas of NH animals that had not received a cochlear implant (see Section “Spiral ganglion cell and peripheral process analysis”). The PBS control animals and the NH animals were close to the black *y* = *x* diagonal line and had a mean PP/SGC ratio of 0.98 and 1.00, respectively, indicating an equal presence of PPs and SGCs for these animals. In all neurotrophin-treated animals, there appeared to be more PP than SGC survival as indicated by the position of the data points above the line, with a mean ratio of 1.26 for the BDNF-treated animals, 1.11 for the NT-3-treated animals, and 1.18 for the Cocktail-treated animals. An ANOVA on these ratios revealed that the high PP/SGC ratios of the neurotrophin groups were not statistically significantly higher than the ratio in the untreated PBS group [*F*_(3,42)_ = 1.2, *p* = 0.32].

In the untreated ear, the PP/SGC ratio of the untreated PBS animals was neatly situated close to the y = x line, with a mean SGC/PP ratio of 1.09. For the neurotrophin-treated groups, the majority of the animals (mean ratios: BDNF, 1.05; NT-3, 1.06; Cocktail, 1.00) closely resemble the ratio of the PBS animals. An ANOVA revealed no difference in ratio between the experimental groups [*F*_(3,42)_ = 0.020, *p* = 0.99]. A ratio slightly larger than 1 (in both treated and untreated ears) may be partly explained by the inclusion in the PP counts of efferent fibers, which make up approximately 2–3% of PPs in the normal-hearing cochlea (e.g., Warren and Liberman, 1989). These efferent fibers, in contrast to SGCs and their PPs, do not degenerate after deafening (e.g., see Figure 6 in Ramekers et al., 2015a).

### Electrically-evoked compound action potential single-pulse results

**FIGURE 5:**
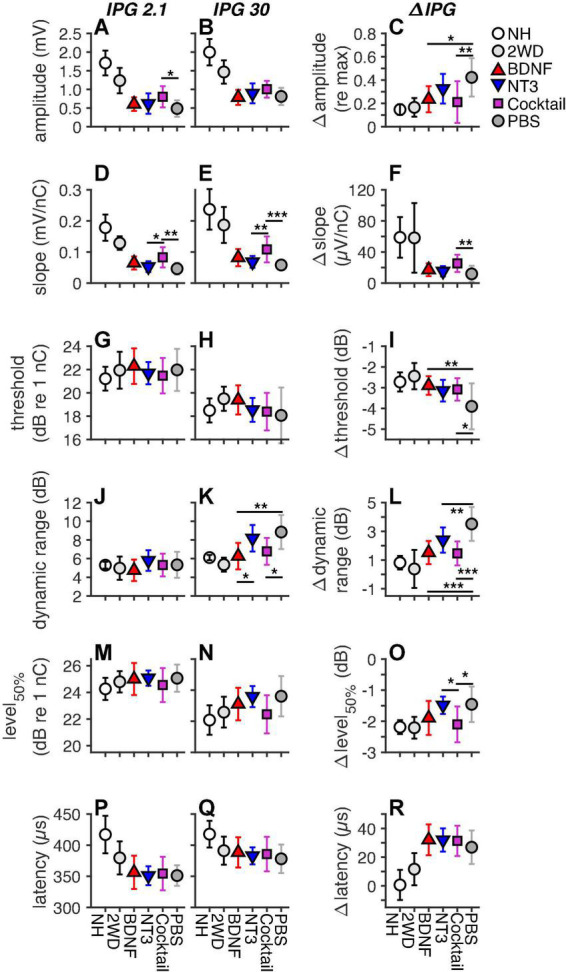
Mean eCAP characteristics for each group, including animals with a deafness duration of 2 weeks (2WD), the time point at which the treatment was started, which were taken from Ramekers et al. (2014). The left and middle columns represent the absolute eCAP characteristics for an IPG of 2.1 and 30 μs, respectively. The right column represents the IPG effect (ΔIPG), as the difference between the two preceding columns. **(A–C)**, maximum amplitude; **(D–F)**, slope; **(G–I)**, threshold; **(J–L)**, dynamic range; **(M–O)**, level_50%_; **(P–R)**, N_1_ latency. NH, *n* = 9; PBS, *n* = 12; BDNF, *n* = 11; NT-3, *n* = 11; Cocktail, *n* = 12. Error bars represent standard deviation. Note that some error bars in the NH group are very small and overlap with the data point. *p < 0.05; **p < 0.01; ***p < 0.001.

The eCAP characteristics derived from the single pulse eCAP recordings are presented in Figure 5, for both IPGs (2.1 and 30 μs) and the IPG effect (ΔIPG). Note that an extra group, 2 weeks deaf (2WD; from Ramekers et al., 2014), was added for the purpose of visualization of the functional status of the auditory nerve at the same duration of deafness, at which the experimental groups received their intervention. The results from these 2WD animals and from the NH animals were not included in the statistical analyses presented in this section.

For the absolute the eCAP characteristic known as maximum amplitude (Figures 5A,B), a statistically significant difference between experimental groups was observed only for an IPG of 2.1 μs (Table 4). Subsequent *post hoc* testing (Table 5) revealed that the Cocktail treatment resulted in a larger maximum eCAP amplitude than the PBS control treatment (Tukey’s HSD, *p* = 0.015). For slope (Figures 5D,E), differences between groups were observed for both IPGs, with the Cocktail-treated animals exhibiting a steeper eCAP slope than the untreated PBS animals (*p*_IPG 2.1_ = 0.0023; p_IPG 30_ < 0.001). Notably, the eCAP slope for the Cocktail animals was also significantly steeper than that for the NT-3-treated animals (*p*_IPG 2.1_ = 0.019; *p*_IPG 30_ = 0.0070). For an IPG of 30 μs, a difference between groups was observed in the dynamic range of the eCAP growth function. Both the BDNF group (*p* = 0.0013) and the Cocktail group (*p* = 0.010) had a narrower dynamic range than the PBS group. The BDNF group also had a narrower dynamic range than the NT-3 treatment group (*p* = 0.028). Finally, a significant group difference was observed for level_50%_, with *post hoc* testing revealing a near-significantly lower level_50%_ for the Cocktail group, as compared to the PBS (*p* = 0.061) or NT-3 groups (*p* = 0.077).

**TABLE 4:** *F*- and *P*-values of the ANOVA on eCAP characteristics of both an IPG of 2.1 and 30 μs, and ΔIPG.

eCAP measure		IPG 2.1 μs	IPG 30 μs	ΔIPG
Amplitude	*F*	3.4	2.1	4.9
	*p*	**0.026**	0.11	**0.0051**
Slope	*F*	5.6	7.2	4.5
	*p*	**0.0025**	**<0.001**	**0.0079**
Threshold	*F*	0.64	1.2	4.5
	*p*	0.59	0.29	**0.0080**
Dynamic range	*F*	1.3	7.0	12.3
	*p*	0.27	**<0.001**	**<0.001**
Level 50%	*F*	0.66	2.9	4.5
	*p*	0.58	**0.045**	**0.0082**
Latency	*F*	0.16	0.45	0.69
	*p*	0.92	0.72	0.56

p-values < 0.05 are shown in bold.

**TABLE 5:** *P*-values from the *post hoc* Tukey’s HSD, following the ANOVA on the eCAP measures for both IPGs.

		IPG 2.1 μs		IPG 30 μs		
Comparison		Amplitude	Slope	Slope	Dynamic range	Level 50%
BDNF–NT-3	*p*	0.99	0.64	0.66	**0.028**	0.73
BDNF–Cocktail	*p*	0.23	0.26	0.12	0.86	0.48
BDNF–PBS	*p*	0.66	0.25	0.19	**0.0013**	0.69
NT-3–Cocktail	*p*	0.29	**0.019**	**0.0070**	0.14	0.077
NT-3–PBS	*p*	0.58	0.91	0.83	0.73	1.0
Cocktail–PBS	*p*	**0.015**	**0.0023**	**<0.001**	**0.010**	0.061

p-values < 0.05 are shown in bold.

When assessing the IPG effect, statistically significant differences between experimental groups (Table 4) were observed for Δamplitude (Figure 5C), Δslope (Figure 5F), Δthreshold (Figure 5I), Δdynamic range (Figure 5L), and level_50%_ (Figure 5O) but not Δlatency (Figure 5R). *Post hoc* testing (Table 6) revealed that Δamplitude and Δthreshold for both BDNF and Cocktail treatment groups was significantly smaller than that for the PBS group. NT-3 treatment also led to a smaller Δthreshold than PBS, which was almost significant (*p* = 0.070). Only for the Cocktail group was Δslope significantly larger than for the PBS group, but still far smaller than for the NH group. All neurotrophic treatments resulted in a significantly smaller and more normal-like Δdynamic range than that of the PBS control group. The Δlevel_50%_ of the Cocktail group was significantly lower than that of the PBS group (*p* = 0.018), and similar to that of the NH animals. Interestingly, the Δlevel_50%_ of the NT-3 animals was similar to that of the PBS group and significantly smaller than that of the Cocktail group (*p* = 0.030).

**TABLE 6:** *P*-values from the *post hoc* Tukey’s pairwise comparison, following the ANOVA on the eCAP measures for ΔIPG.

Comparison		Amplitude	Slope	Threshold	Dynamic range	Level 50%
BDNF–NT-3	*p*	0.50	0.96	0.85	0.15	0.25
BDNF–Cocktail	*p*	0.98	0.17	0.92	1.0	0.77
BDNF–PBS	*p*	**0.022**	0.53	**0.0086**	**<0.001**	0.19
NT-3–Cocktail	*p*	0.27	0.060	1.0	0.10	**0.030**
NT-3–PBS	*p*	0.41	0.82	0.070	**0.033**	1.0
Cocktail–PBS	*p*	**0.0064**	**0.0053**	**0.037**	**<0.001**	**0.018**

p-values < 0.05 are shown in bold.

#### Inter-phase gap effect and spiral ganglion cell survival

**FIGURE 6:**
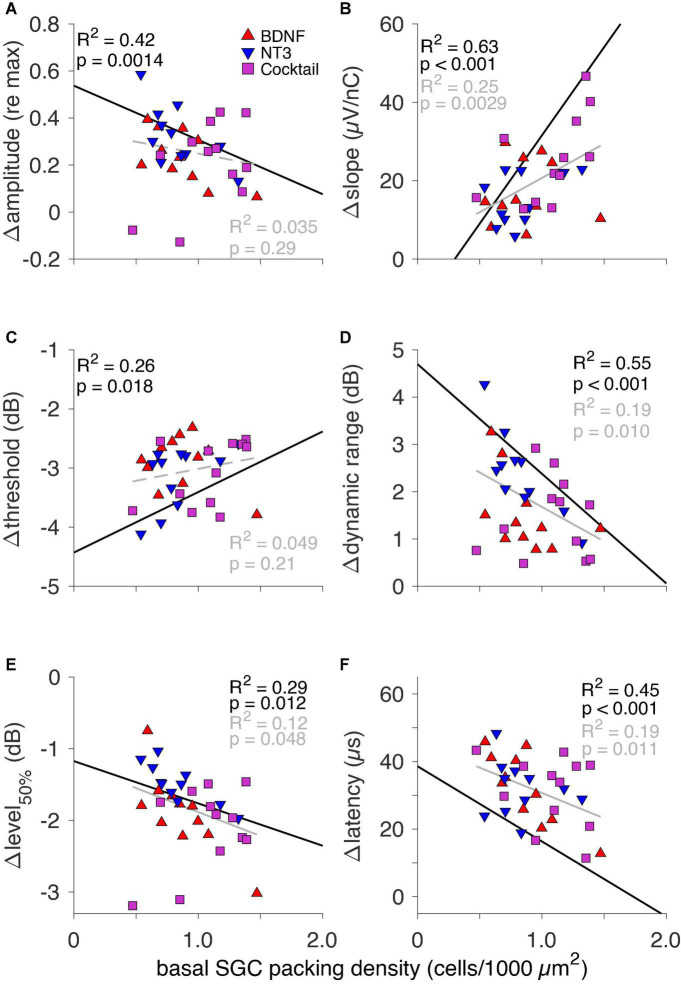
The IPG effect for all six eCAP characteristics as a function of basal SGC packing density for individual neurotrophin-treated animals only. Black text and regression lines represent the correlation between the ΔIPG measure and SGC packing density for both NH and the sham-treated PBS (i.e., non-neurotrophin-treated) groups. The gray text and regression lines represent this correlation for the neurotrophin-treated animals only. Solid lines indicate an *R*^2^ value with *p* < 0.05; dashed lines indicate *p* > 0.05. **(A)** Δamplitude, **(B)** Δslope, **(C)** Δthreshold, **(D)** Δdynamic range, **(E)** Δlevel_50%_, and **(F)** Δlatency.

The eCAP IPG effects as a function of SGC packing density are shown in Figure 6 for the three neurotrophin-treated groups. For the NH and PBS groups there was a significant correlation between each ΔIPG measure and SGC survival, as indicated by the black lines in Figure 6 (data points not shown). In these groups Δamplitude (Figure 6A, *R*^2^ = 0.42; *p* = 0.0014), Δthreshold (Figure 6C, *R*^2^ = 0.26; *p* = 0.018), Δdynamic range (Figure 6D, *R*^2^ = 0.55; *p* < 0.001), Δlevel_50%_ (Figure 6E, *R*^2^ = 0.29; *p* = 0.012), and Δlatency (Figure 6F, *R*^2^ = 0.45; *p* < 0.001) all decreased, while Δslope (Figure 6B, *R*^2^ = 0.63; *p* < 0.001) increased, with more SGC survival. When looking at the same relationship in the neurotrophin-treated animals, the relationship between Δamplitude and Δthreshold with SGC survival became less clear and not significant, but the values of both these measures for the individual animals gravitated more toward the NH than the PBS values. For Δslope, Δdynamic, Δlevel_50%_, and Δlatency, a significant correlation between the ΔIPG measure and SGC survival remained present after neurotrophic treatment, albeit with a smaller effect size. For Δdynamic range, the majority of the neurotrophin-treated animals, predominantly BDNF-treated or Cocktail-treated, exhibited values close to NH, as indicated by their position below the black regression line. For both Δslope and Δlatency, most animals were more inclined toward the PBS-treated values. Δlevel_50%_ of the neurotrophin-treated animals closely followed the black regression line, i.e., this response measure seems to reflect neural survival.

### Electrically-evoked compound action potential masker-probe results

**FIGURE 7:**
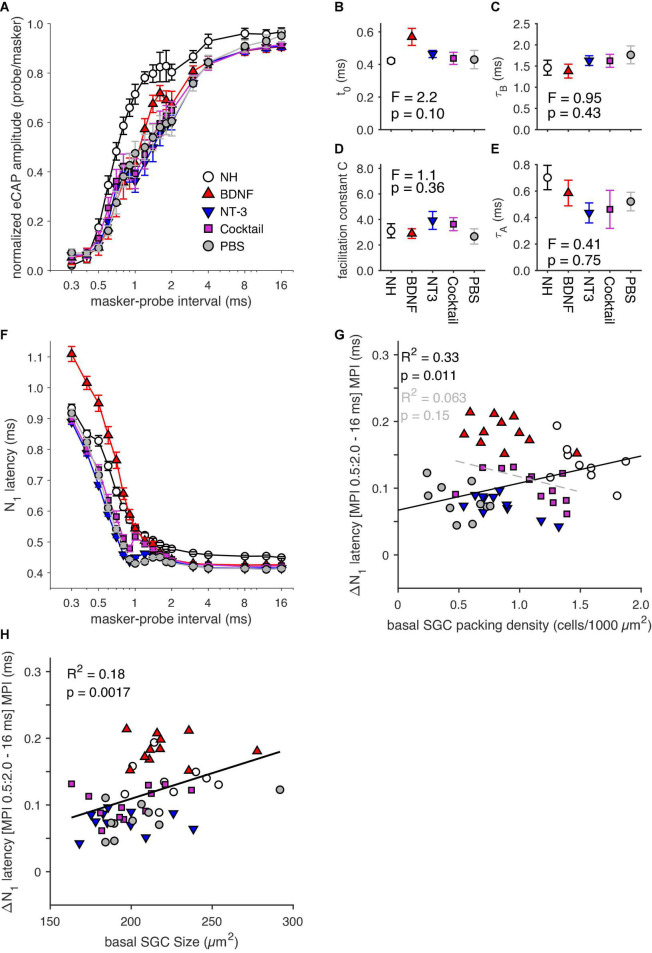
**(A)** Group means of masker-probe recovery functions constructed using 18 masker-probe intervals (MPIs; range: 0.3–16 ms). **(B–E)** Fitting parameters derived from fitting a double exponential to the recovery functions in panel **(A)**. **(F)** N_1_ latency of the eCAP evoked by the probe stimulus for the 18 MPIs. **(G)** ΔN_1_ latency between the mean latency of the MPI range 0.5–2 ms, and that for the 16-ms MPI for individual animals as a function of basal SGC packing density. **(H)** ΔN_1_ latency between the mean latency of the MPI range 0.5–2 ms, and that for the 16-ms MPI for individual animals as a function of basal SGC size. NH, *n* = 9; PBS, *n* = 10; BDNF, *n* = 11; NT-3, *n* = 11; Cocktail, *n* = 12. Error bars represent SEM.

Group means of normalized amplitudes of probe-evoked eCAPs as a function of MPI are shown in Figure 7A. These recovery functions had a non-monotonic shape for all groups, but the PBS control group. A local maximum was at an MPI of approximately 1.6 ms for the NH and BDNF animals, suggesting a more normal-like recovery after deafening, following BDNF treatment. For the NT-3, and Cocktail groups, this local peak was around 0.8-ms MPI. As the MPI ranging from 0.5 to 2 ms showed the most differential effects on the normalized eCAP amplitude, an LMM analysis was performed on the amplitudes with MPI within the 0.5–2 ms range as the repeat to investigate the effects of treatment. This revealed no significant main effect of treatment [*F*_(3,78.7)_ = 2.2, *p* = 0.096], but an interaction effect between treatment and MPI was observed [*F*_(3,194.0)_ = 7.8, *p* < 0.001]. This interaction was caused by the BDNF group [*t*_(194.0)_ = 3.5, *p* < 0.001] that, as stated above and seen in Figure 7A, had its local maximum at a higher MPI than the other experimental groups.

A double exponential fit was performed on the recovery functions (see Section “Masker-probe recordings,” including Equation 2), from which the fitting parameters can provide additional insight into the effects of the neurotrophin treatment. The absolute refractory period (Figure 7B) appeared to be longer for the BDNF group than for the other experimental groups and the NH group, but an ANOVA revealed no statistically significant difference between experimental groups [*F*_(3,40)_ = 2.2, *p* = 0.10]. Figure 7C shows slightly longer recovery time constants (τ_B_) for the NT-3, Cocktail, and PBS groups than the NH groups, whereas the BDNF group shows a similar recovery time to the NH animals. However, no differences were found between experimental groups for τ_B_ [*F*_(3,40)_ = 0.95, *p* = 0.43]. For both the facilitation constant *c* and τ_A_ (Figures 7D,E), the BDNF group appeared the most normal-like of the neurotrophin groups, whereas both the NT-3 and Cocktail groups appeared to differ the most from the NH group. However, the differences between experimental groups were not statistically significant [*F*_C(3,40)_ = 1.1, *p* = 0.36; *F*_τ A(3,40)_ = 0.41, *p* = 0.75].

The eCAP N_1_ latency (Figure 7F) increased with decreasing MPI. For all groups, this increase was especially steep for MPIs smaller than 0.8 ms. For the BDNF-treated animals this increase in latency was most prominent, exceeding the NH levels, while the latency for both other neurotrophin-treated groups was shorter than for the NH group and on par with the PBS control group. Interestingly, the eCAP latency for the Cocktail treatment group followed the same increase in latency with decreasing MPI as the BDNF treatment group for MPIs above 1 ms, before crossing over to values observed for NT-3 and PBS groups for MPIs below 1 ms. An LMM using the same MPI range as for the amplitude analysis was performed on the latency data. The model indeed revealed a treatment effect [*F*_(3,285.5)_ = 12.5; *p* < 0.001], with an interaction between treatment and MPI [*F*_(4,252.8)_ = 12.2, *p* < 0.001], indicating a strong relationship between the two. To further investigate this relationship, ΔN_1_ latency was calculated by taking the difference between the mean N_1_ latency for the MPI range of 0.5 to 2 ms and the N_1_ latency from the 16 ms MPI. Figure 7G shows this ΔN_1_ latency for individual animals as a function of SGC survival, clearly illustrating larger values for the BDNF-treated animals (∼0.2 ms) than the other experimental animals (∼0.1 ms). An ANOVA confirmed a significant effect of treatment between experimental groups [*F*_(3,40)_ = 3.4, *p* = 0.027], with *post hoc* tests indicating a near-significantly longer ΔN_1_ latency for the BDNF animals as compared to the PBS animals (*p* = 0.056) and a significantly longer ΔN_1_ latency for the BDNF animals than for the NT-3 animals (*p* = 0.034). When looking at the NH and PBS control animals, there is a significant positive correlation between ΔN_1_ latency and SGC packing density (*R*^2^ = 0.33; *p* = 0.011). Interestingly, this relationship disappears following neurotrophic treatment (Figure 7G, gray dashed line).

As cell size may influence stimulus response latency (discussed by Ramekers et al., 2015a), we also investigated a possible relationship between ΔN_1_ latency and SGC perikaryal area (Figure 7H), which revealed moderate but statistically significant positive correlations for both the NH and PBS groups and for the neurotrophin-treated groups. In addition, a multiple regression analysis was performed for the experimental groups to determine if treatment, SGC packing density, and/or SGC size can predict ΔN_1_ latency. A statistically significant regression model was found [*F*_(1,42)_ = 5.3, *p* = 0.27; *R*^2^ = 0.11], with treatment as a statistically significant predictor (*t* = 2.3, *p* = 0.027), but not SGC packing density (*t* = 0.75, *p* = 0.46) or cell size (*t* = 0.16, *p* = 0.88).

### Electrically-evoked compound action potential pulse-trains

#### First 10 pulses

**FIGURE 8:**
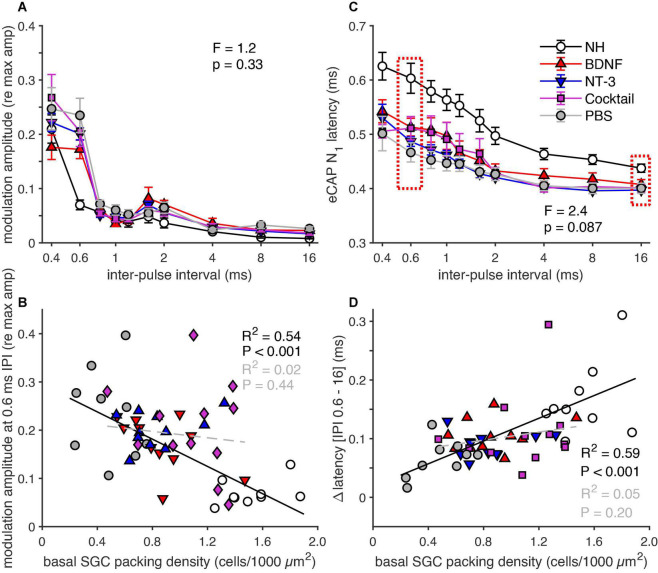
eCAP parameters derived from the responses to the first ten pulses in a pulse train. **(A)** Group means of the normalized eCAP amplitude modulation, of the last six of the ten pulses, as function of IPI. **(B)** Group means of eCAP N_1_ latency of the last six of the ten pulses, as function of IPI. **(C)** Amplitude modulation for an IPI of 0.6 ms for all individual animals as a function of basal SGC packing density, regression was calculated on the NH and PBS animals (black line) and on the neurotrophin-treated animals (gray line). **(D)** ΔN_1_ latency between 0.6 and 16 ms, for the individual animals as a function of SGC packing density. The regression was calculated on the NH and PBS animals (black line) and on the neurotrophin-treated animals (gray line). NH, *n* = 9; PBS, *n* = 9; BDNF, *n* = 11; NT-3, *n* = 11; Cocktail, *n* = 12. Error bars represent SEM.

The normalized amplitude modulation (as obtained from the alternating N_1_-P_2_ amplitude shown in Figures 1E,G) and the eCAP N_1_ latency, following the first ten pulses in a pulse train, are shown in Figure 8. The amplitude modulation (Figure 8A) was quite similar for all groups at IPIs of 0.8 ms or longer but nearing the refractory period the modulation of the deafened animals deviated strongly from the NH animals, especially at 0.6 ms. At this IPI, the NH animals only showed a weak modulation (∼0.07), and the PBS group showed the largest amplitude modulation of all experimental groups (∼0.25), whereas the BDNF group showed the smallest modulation of these groups (∼0.17). An ANOVA revealed, however, no significant difference between the experimental groups for an IPI of 0.6 ms [*F*_(3,39)_ = 1.2, *p* = 0.33].

Figure 8B shows the normalized amplitude modulation as a function of SGC survival for each animal of every group. A strong and significant negative correlation was observed between modulation and survival when looking at both the NH and PBS group (Figure 8B, black line). This relationship did not persist following neurotrophic treatment, as indicated by the neurotrophin-treated groups mostly showing similar amplitude modulation to the PBS control animals, regardless of SGC packing density, with only a few animals showing a shallow normal-like modulation. No significant correlation between amplitude modulation and SGC survival was observed in these animals (Figure 8B, gray dashed line).

While the N_1_ latency, shown as the mean latency of the eCAPs of the final six of the ten pulses in Figure 8C, increased with decreasing IPI in all guinea pigs, this latency increase was smaller for the experimental groups than for the NH animals. Both the BDNF and the Cocktail group exhibited the most normal-like N_1_ latency of the eCAP elicited by the pulses with the IPI paired with the highest amplitude modulation (0.6 ms). This was further explored by analyzing the ΔN_1_ latency (N_1_ latency difference between the IPI of 0.6 ms and 16 ms; Figure 8C, red dashed boxes), which revealed a close-to-significant difference between the experimental groups [ANOVA; *F*_(3,39)_ = 2.4, *p* = 0.087], hinting toward a treatment effect. The ΔN_1_ latency positively correlated with SGC survival in both PBS and NH animals (Figure 8D, black line). Similar to the amplitude modulation, a clear relationship between ΔN_1_ latency and SGC survival was absent. Some animals showed an ΔN_1_ latency quite similar to the NH animals, while others more closely resembled the PBS animals, independent of cellular survival. No significant correlation was observed for the animals of all three neurotrophin-treated groups (Figure 8D, gray dashed line).

#### Last 10 pulses

**FIGURE 9:**
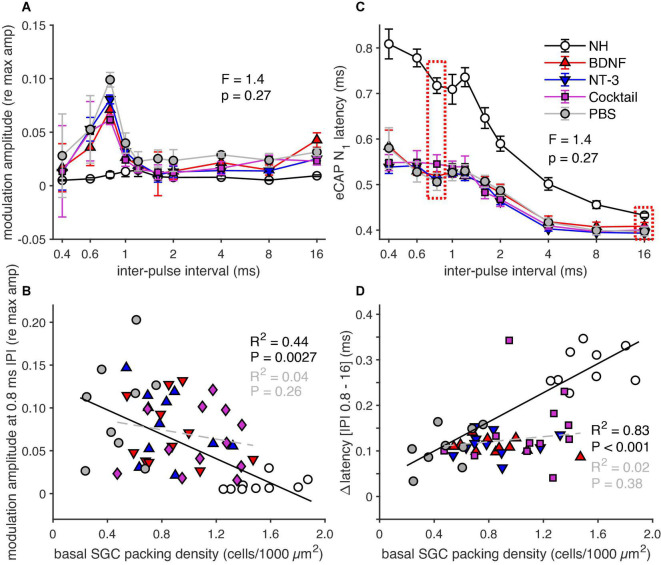
eCAP parameters derived from the responses to the last ten pulses of a 100-ms pulse train. **(A)** Group means of the normalized eCAP amplitude modulation as a function of IPI for all ten pulses. **(B)** Group means of eCAP N_1_ latency as a function of IPI for all ten pulses. **(C)** Amplitude modulation for an IPI of 0.8 ms for all individual animals as a function of basal SGC packing density, regression was performed on the NH and PBS animals only. **(D)** ΔN_1_ latency between 0.8 and 16 ms, for the individual animals as a function of SGC packing density. The regression was calculated on the NH and PBS animals only. NH, *n* = 9; PBS, *n* = 9; BDNF, *n* = 11; NT-3, *n* = 11; Cocktail, *n* = 12. Error bars represent SEM.

Figure 9A shows the normalized amplitude modulation as observed for the last ten pulses in a 100-ms pulse train. The modulation largely displayed the same group differences as in response to the first ten pulses (Figure 8A), in that modulation was observed mainly in the experimental groups, and hardly in the NH group. In addition, the magnitude of amplitude modulation had decreased substantially (to ∼0.1 for PBS, to ∼0.01 for NH) as compared to the responses to the first ten pulses, in line with the more adaptive state at the end of the pulse train. The largest amplitude modulation in the experimental groups was observed at an IPI of 0.8 ms, with the Cocktail-treated animals showing the least amplitude modulation. However, this effect was not significant as an ANOVA revealed no differences between the experimental groups [*F*_(3,39)_ = 1.4, *p* = 0.27]. The amplitude modulation at an IPI of 0.8 ms, correlated significantly with SGC survival (Figure 9B; *R*^2^ = 0.44, *p* = 0.0027) in the NH and PBS groups. This relationship was not observed in the neurotrophin-treated groups (*R*^2^ = 0.04, *p* = 0.26), in which some animals followed the Δamplitude modulation seen in PBS animals, while others approached the NH situation.

For the last ten pulses, the N_1_ latency (Figure 9C) was longer for IPIs < 2 ms with a greater difference between the NH and experimental groups than at the start of the pulse train (Figure 8C). This increase in N_1_ latency with decreasing IPI was larger for the last ten pulses than for the first ten pulses. When looking at the ΔN_1_ latency (difference in N_1_ latency between the IPI with the highest amplitude modulation, 0.8 ms, and 16 ms), there was no significant difference between the experimental groups [*F*_(3,39)_ = 1.4, *p* = 0.27]. Where a strong positive correlation was observed between ΔN_1_ latency and SGC packing density for the NH and PBS groups (Figure 9D), this relationship did not persist for the neurotrophin-treated animals. The ΔN_1_ latency of nearly all neurotrophin-treated animals was situated below the black regression line; thus, resembling the ΔN_1_ latency of the PBS animals, seemingly unaffected by the corresponding SGC packing density.

## Discussion

In the present study, we investigated the preservative effects of the neurotrophins BDNF, NT-3, and a combination thereof on the auditory nerve in ototoxically deafened guinea pigs delivered by gelatin sponge onto the RWM. We hypothesized that treatment with NT-3 would yield better apical survival than BDNF treatment, whereas a cocktail of both neurotrophins could yield a synergetic effect that might outperform that of BDNF alone. Contrary to this hypothesis, we found that treatment with NT-3 did not lead to more apical SGC survival than BDNF treatment. Additionally, in accordance with our hypothesis, we did observe superior cellular preservation, following Cocktail treatment in the basal turn and more survival in the middle and apical turns.

### Neurotrophin-mediated survival of spiral ganglion cell soma and peripheral processes

Treatment with BDNF resulted in significantly enhanced survival of SGCs in the cochlear base compared to both the contralateral untreated ears and to untreated PBS animals, confirming our previous findings with the same method (Havenith et al., 2011, 2015). The reason for choosing this method rather than for instance the implantable mini-osmotic pump (e.g., Miller et al., 1997; Ramekers et al., 2015a) was twofold: first, applying gelatin sponge to the RWM is clinically feasible and therefore provides room for translational studies in humans; and second, with a treatment effect limited to the cochlear base with BDNF alone (Havenith et al., 2011, 2015), there is ample room for improvement when using different neurotrophins or a combination thereof. While we appreciate drug delivery *via* gelatin sponge is based on passive diffusion and is, therefore, not as controlled as for instance a direct infusion, there is no reason to assume there might have been major differences in diffusion speed between the two structurally homogenous neurotrophins.

Treatment with the neurotrophin NT-3 has been shown to yield similar results as with BDNF when applied with an osmotic pump (Ernfors et al., 1996; Staecker et al., 1996; Gillespie et al., 2004). However, similar to the findings by Miller et al. (1997), the protective effect of NT-3 in the present study was smaller (and statistically non-significant) than with BDNF. While the receptors for both BDNF (TrkB) and NT-3 (TrkC) are reportedly co-expressed in the cochlea (Ylikoski et al., 1993), this has not been thoroughly quantified throughout the cochlear turns in the adult cochlea (Fritzsch et al., 2016). Therefore, given the gradients that exist in the cochlea, i.e., BDNF expression being higher in the base and NT-3 expression higher in the apex (Davis, 2003 [Review]), SGC survival with either one neurotrophin may follow that same gradient. Unexpectedly, we observed a conspicuously similar survival pattern across the cochlea for both treatment groups: higher SGC survival in the base but no obvious effect of NT-3 beyond the basal turn. This, in turn, is more in line with our previous studies, showing a predominantly basal SGC preservation, following gelatin-sponge-mediated BDNF delivery (Havenith et al., 2011, 2015; Vink et al., 2020). Finally, as BDNF is naturally more expressed in the basal region of the cochlea, it stands to reason that these cells exhibit less sensitivity to NT-3.

A combined application of BDNF and NT-3 has been performed previously *in vitro* (Mou et al., 1997; Needham et al., 2012; Wright et al., 2016) and *in vivo* (Staecker et al., 1996; Richardson et al., 2005; Wise et al., 2005; Landry et al., 2011). Both Staecker et al. (1996) and Mou et al. (1997) directly compared the effect of this cocktail to that of BDNF and NT-3 separately. Mou et al. (1997) observed a much stronger effect on SGC survival following combined treatment with BDNF and NT-3 than with either one separately, indicating a synergy between the two neurotrophins. Staecker et al. (1996) reported similar SGC preservation between cocktail-treated animals and NT-3-treated animals – both of which slightly higher than with BDNF treatment. Taking into account the reported synergistic relationship between BDNF and NT-3 by Mou et al. (1997) and a possible ceiling effect in the study by Staecker et al. (1996), we hypothesized that in the present study, the Cocktail treatment would lead to better SGC preservation than with either of neurotrophin separately. A comparison of the mean absolute survival in the treated right ears indeed confirmed that the Cocktail treatment was more potent in preventing SGC and PP loss than BDNF or NT-3 separately, even extending the neuroprotection to the upper middle and apical turns in comparison to BDNF. However, this initially straightforward finding is complicated by the SGC and PP counts in the untreated contralateral ears of these animals, which were higher for the Cocktail-treated animals. Within-subject comparison of SGC survival across ears should yield a more accurate estimation of treatment effect since it cancels out irrelevant across-subject variability, such as natural SGC presence, or susceptibility to the systemic ototoxic treatment. Presented as such (Figure 4A), the data favor the BDNF treatment over the Cocktail treatment for both SGCs and their PPs. Which of the two analyses more accurately reflects treatment efficacy depends largely on what caused the higher SGC packing density in the contralateral ears of the Cocktail-treated animals.

One possible explanation for higher SGC counts would be that the deafening procedure was less effective in the Cocktail-treated animals, so that a larger residual population of hair cells more potently supported SGC survival. However, hair cell counts and ABR threshold shifts indicated that there were no differences among groups (Table 1) as intended by randomization. Second, since the animals of every batch of experimental animals were randomly divided into one of the treatment groups, the observed survival cannot be attributed to an anomalous single batch of animals. A third explanation could be that BDNF, and NT-3 diffused from the right cochlea, most likely *via* the cochlear aqueduct and the cerebrospinal fluid, to the left ear (as proposed by Salt et al., 2016). In a study on the distribution of radio-labeled human NT-3 injected into the scala tympani in guinea pigs, Richardson et al. (2019) sporadically observed thus labeled NT-3 in the contralateral cochlea. Similarly, Lee et al. (2020) investigated intratympanic nanoparticle-conjugated gentamicin delivery in the rat and found their nanoparticles in the outer hair cells of the contralateral cochlea, mainly in the basal turn, in four out of six animals. Hence, the possibility of neurotrophins delivered to one ear affecting SGC survival in the contralateral ear appears quite viable. The observation that enhanced SGC survival in the contralateral ear only occurs with the combination of BDNF and NT-3 may then reflect the synergetic effect of the two naturally present neurotrophins in the healthy cochlea. It is important to note that since the size and nature of the human cerebrospinal fluid compartments and volume dynamics, it is unlikely to observe such an effect in humans.

Conversely, SGC survival in the contralateral ears of the BDNF-treated animals was lower than observed in the contralateral ears of the other experimental groups and in the treated ears of the PBS control group, which also could not be accounted for by hair cell counts, ABR thresholds or batch effects (see above). We do not have an explanation for this particular finding.

In short, the Cocktail treatment resulted in the highest neural survival in both the treated and the untreated ear. Since there is no other satisfactory explanation for this observation than the mere effect of the treatment, we conclude that the Cocktail treatment is preferred in terms of neural survival.

### Equal preservation of the spiral ganglion cell soma and its peripheral process

In the untreated PBS animals, the PP/SGC ratio remained close to 1 (Figures 4C,D), indicating simultaneous degeneration of both neural elements. This observation confirms similar findings in a larger group of animals with varying durations of deafness (Ramekers et al., 2020). The ratio for the neurotrophin-treated animals was on average slightly higher than 1, indicating that at least each surviving SGC soma possessed a PP. This finding contradicts our previous observations in guinea pigs receiving BDNF *via* an osmotic pump, in which preservation of the SGC somata was more effective than that of their PPs (Vink et al., 2021). This discrepancy may be explained by the difference in delivery method: the osmotic pumps infused BDNF directly into the cochlea, leading to significant protection of SGCs from base to apex, whereas in the present study the RWM application only resulted in partial preservation in the base. Possibly, a relatively high concentration is needed throughout the cochlea to reach and subsequently protect the SGC soma through the modiolar wall, while lower concentrations may be sufficient to preserve the PPs protruding through the spiral lamina toward the neurotrophin-rich scalae.

An important additional observation is that the present data do not show an obvious difference in PP preservation with BDNF, NT-3, or a cocktail of both (Figures 3, 4). This finding is in line with a recent *in vitro* study showing that increasing concentrations of either BDNF or NT-3 yield very similar results on total neurite outgrowth or mean neurite length in murine spiral ganglion explants (Rousset et al., 2022). However, others have reported that BDNF results in more complexity (among others, more neurite outgrowth) while NT-3 results in longer neurites (Xie et al., 2013). In the present *in vivo* model, we have not assessed PP length or complexity, but it is likely that *in vivo* PP outgrowth is much more restrained by the physical boundaries in the cochlea (e.g., bone and fluid compartments), so that the focus is on the preservation of existing neurites rather than on outgrowth.

### Effects on auditory nerve responsiveness

Studies on neurotrophic treatment of the auditory nerve have mainly focused on mere numerical survival of SGCs, only occasionally accompanied by functional measures, such as electrically evoked ABRs (see Ramekers et al., 2012 for a review) and, only more recently, eCAPs (Ramekers et al., 2015a; Vink et al., 2020). For example, Landry et al. (2011) reported lower eABR thresholds for neurotrophin-treated animals than for control animals or normal-hearing controls. However, since the main reason for SGC preservation is to maintain a healthy and functional neural interface for CIs, equal attention should be paid to functional SGC preservation in terms of proper responsiveness to electrical stimulation. Whereas the suitability of a neurotrophin in mediating SGC survival is easily assessed by cell counts, its suitability in terms of functional preservation is much more complicated. Adamson et al. (2002) showed that treatment with BDNF or NT-3 of murine SGCs *in vitro* gives spectacularly different response properties to electrical stimulation: BDNF leads SGCs to fire phasically (as occurs naturally in the cochlear base), whereas SGCs treated with NT-3 fire tonically (as in the apex). Therefore, although both neurotrophins potently prevent SGC degeneration (as discussed above), their suitability in preserving the nerve for optimal CI usage depends on which type of electrical responsiveness is required.

In the present study, the effect of both neurotrophins on the eCAP, separately and a cocktail of both, was assessed using several stimulation protocols, designed to examine different aspects of neural responsiveness to electrical stimulation. eCAPs evoked by single-pulse stimuli have been shown to be informative of the condition of the SGC population (Prado-Guitierrez et al., 2006; Ramekers et al., 2014, 2015a; Schvartz-Leyzac et al., 2019, 2020; Vink et al., 2020). Although the absolute eCAP measures showed some improvement for the Cocktail group compared to the untreated PBS group (steeper slope of the input-output function, accompanied by a narrower dynamic range), it has been shown that a more accurate prediction of the neural condition can be obtained with relative measures, such as the IPG effect. Accordingly, for five of the six tested IPG effects, the Cocktail group exhibited more normal-like responses that were significantly different from the untreated PBS animals. Based on these IPG effects, the combination of BDNF and NT-3 was clearly superior to either one separately, while the BDNF-treated animals more often differed from the untreated PBS animals than the NT-3-treated animals. These findings appear to accurately reflect the histological assessment of the treatment effect of the implanted right ears, being that the Cocktail provided the most protection, followed by BDNF alone. However, we have previously shown that these IPG effects are not simply reflections of SGC survival since BDNF can improve these measures beyond the level corresponding to the animal’s SGC count (Ramekers et al., 2015a). Rather, these measures reflect what has been termed “cochlear health” – which is an as yet undefined combination of number and condition of the surviving SGCs. Judging by the IPG data in relation to cellular survival shown in the present study, we could argue that Δamplitude, Δslope, and Δlevel_50%_ can be indicators of the number of surviving cells, while Δthreshold, Δdynamic range, and Δlatency indicate the more functional preservation of the SGCs. The IPG effects presented here, thus, indicate that the cocktail of BDNF and NT-3 improved cochlear health more than either of the neurotrophin separately.

Since sound encoding with CIs involves high-frequency pulsatile stimulation, proper temporal responsiveness of the SGC population to consecutive pulses is crucial. The final two eCAP recording paradigms were, therefore, aimed to characterize the effects of the three treatments on temporal responsiveness. Fitting of the two-pulse masker-probe recovery functions did not reveal statistically significant differences between groups, although the BDNF-treated animals appeared to have a longer absolute refractory period *t*_0_ (Figure 7B), as seen before in Ramekers et al. (2015a, their Figure 11A). This is in line with their phasic properties, as they should be able to fire fast but can take longer to recover. An opposite effect, i.e., a shorter *t*_0_ following NT-3 treatment, was not observed. A separate, apparently slowing, effect of BDNF, i.e., longer N_1_ latency of the eCAP evoked by the probe stimulus (Figure 7F), was statistically significant. Since BDNF increases cell size (e.g., Agterberg et al., 2008; Van Loon et al., 2013; Ramekers et al., 2015a [all in guinea pig]; Leake et al., 2011 [cat]), there was a possibility that a secondary effect of a larger cell is that action potentials take longer to cross the soma. A significant positive correlation was observed between the increase in N_1_ latency for short MPIs and SGC size for both the NH and PBS animals and the neurotrophin-treated groups in the present study, but this only explained a modest proportion of the exaggerated latency increase following BDNF treatment (Figure 7H). It remains to be determined whether this apparent slowing down of SGC responsiveness by BDNF would significantly affect CI function. Nevertheless, these results indicate that by using a cocktail of both BDNF and NT-3 (or NT-3 separately) any delays in responsiveness can be avoided. These results are somewhat in line with results from Wright et al. (2016), who investigated the *in vitro* firing properties of SGCs in rat spiral ganglion cultures following the combined treatment of BDNF and NT-3. They found that, although each separate neurotrophin may exert functional changes in activity, exposure to a combination of the two maintains the existing neuronal firing properties.

We had expected that especially eCAP responses to pulse trains would be different for BDNF-treated and NT-3-treated animals, given the aforementioned results by Adamson et al. (2002). However, both at the onset of the pulse train (Figure 8) and at the end of a 100-ms pulse train (Figure 9), the differences between the experimental groups in terms of refractory behavior and latency were negligible. The findings are somewhat in accordance with the data observed in one of our previous studies. Ramekers et al. (2015a) showed that treatment with BDNF, by means of an osmotic pump, had a large effect on SGC survival throughout the cochlea paired with a clear IPG effect. There was, however, only a significant difference in amplitude modulation between NH and 14-weeks deaf control animals and the BDNF-treated animals situated in between, analogous to the amplitude modulation distribution of the neurotrophin-treated animals in the present study (Figures 8A, 9A).

Taken together, treatment with the cocktail of BDNF and NT-3 improved cochlear health, more so than each component separately, as exemplified by the IPG effects. However, the effects of neurotrophic treatment on the masker-probe and pulse-train responses were limited, indicating that these responses are less sensitive to changes in neural health than the IPG effects.

## Conclusion

In the present study, we investigated the preservative effects of a combined treatment of BDNF and NT-3 on the auditory nerve in terms of both cellular survival and electrophysiological function. The Cocktail treatment resulted in the strongest effect on cellular survival in the treated ear, whereas treatment with BDNF alone resulted in the highest survival compared to the untreated contralateral ear. The Cocktail treatment resulted in the most normal-like eCAPs to a single pulse (IPG effects) of all the experimental groups. None of the neurotrophin treatments stood out in the neural recovery and pulse-train results. The finding that neural survival was higher in the untreated contralateral ear of the Cocktail group than in the other treatment groups was unexpected. This observation complicated the use of the untreated contralateral ear as an internal control. Therefore, we are unable to decisively conclude whether BDNF or a cocktail of BDNF and NT-3 is the preferred treatment. The proposed explanation of a contralateral effect would favor the Cocktail treatment over BDNF alone, although a thorough bilateral cochlear sampling study is warranted to prove this hypothesis. Since the Cocktail treatment also resulted in the most normal-like eCAP responsiveness and given that both BDNF and NT-3 are expressed in the healthy adult cochlea, we propose that a combination treatment of both BDNF and NT-3 is preferable to either neurotrophin alone. Other experimental delivery and/or recording methods may be required to shed more light on the effects of the combined treatment on the function of the auditory nerve.

## Data availability statement

The raw data supporting the conclusions of this article will be made available by the authors, without undue reservation.

## Ethics statement

The animal study was reviewed and approved by the Dutch Central Authority for Scientific Procedures on Animals (CCD: 1150020174315).

## Author contributions

DR, HV, and HT contributed to the conceptualization. DR and HV contributed to the methodology and supervision. DR and HAV contributed to the software, investigation, and formal analysis. HAV contributed to the data curation and visualization. HAV, DR, and HV contributed to the writing—original draft. HAV, DR, HV, and HT contributed to the writing—revisions and editing. All authors have contributed to the article and approved the submitted version.
